# Navigating the Path to Food Security in China: Challenges, Policies, and Future Directions †

**DOI:** 10.3390/foods14040644

**Published:** 2025-02-14

**Authors:** Mingyuan Du, Jiaqiang Lei, Shengyu Li

**Affiliations:** National Engineering Technology Research Centre for Desert-Oasis Ecological Construction, The Xinjiang Institute of Ecology and Geography, Chinese Academy of Sciences, Urumqi 830011, China; leijq@ms.xjb.ac.cn (J.L.); oasis@ms.xjb.ac.cn (S.L.)

**Keywords:** food security, challenges, strategies, sustainability, agriculture, environment

## Abstract

This paper provides a comprehensive review and an in-depth analysis of the multifaceted issues surrounding food security in China, exploring historical trends, current challenges, and future strategies. Drawing upon a wide range of sources including government reports, the academic literature, and expert analyses, it examines the complex interplay of factors influencing food production, distribution, and consumption in China. The paper highlights the importance of addressing environmental sustainability, technological innovation, and social equity in shaping China’s food security agenda. By synthesizing key findings and proposing actionable recommendations, this paper contributes to the ongoing discourse on food security in China and offers insights for policymakers, researchers, and practitioners alike. These findings underscore the need for integrated policies that promote sustainable agricultural practices, technological innovation, and infrastructure development while supporting smallholder farmers, ensuring that China’s food security remains resilient in the face of climate change and evolving global food dynamics.

## 1. Introduction

China’s food security is not merely a national issue; it has significant implications for global food markets, trade, and environmental sustainability. In 1995, Lester R. Brown [1] highlighted the potential for a looming food crisis in his seminal work “Who Will Feed China?”. By 2004, China had transitioned from a net food exporter to a net importer, bringing its food challenges into sharper global focus. Since then, discussions surrounding China’s food supply have intensified, including debates on the so-called “China food threat theory”, which explores how the nation’s food security dynamics impact the global economy and food systems [2,3,4].

In the face of global climate change, achieving sustainable food production is a critical challenge, particularly for countries with large populations like China. With nearly 20 percent of the global population but less than 10 percent of the world’s arable land and only 6 percent of its freshwater resources, China’s food security challenges are uniquely pressing [5,6]. This imbalance between population size and natural resource availability underscores the importance of understanding and addressing China’s food production and supply issues, and the question of “who will feed China” remains timely [7].

China’s food security is a multifaceted issue encompassing agriculture, economics, public health, and sociopolitical stability [8]. Numerous studies have assessed its food security from varying perspectives, such as food availability, access, utilization, and resilience to food insecurity [6,9,10,11,12,13,14,15,16,17]. These evaluations have laid the groundwork for identifying actionable strategies to improve China’s food systems. In recent years, researchers have increasingly focused on emerging solutions, such as enhancing resilience through traditional farming practices and organic methods that adapt to climate challenges [18,19]. Concurrently, innovations like precision agriculture, biotechnology, and climate-smart techniques are viewed as pivotal for coping with environmental changes and boosting productivity [20,21].

Beyond technological advances, policy interventions are vital. Well-designed policies and incentives that promote sustainable farming, reduce greenhouse gas emissions, and support smallholder farmers are critical to maintaining food security in the context of a warming climate [22]. Additionally, air quality improvements have been identified as a potential factor for enhancing agricultural productivity and food security in China [23]. These interconnected challenges and solutions place China at the center of global food security discussions.

This review responds to the growing international focus on China’s agricultural and food security issues by synthesizing the existing literature to provide a comprehensive analysis of recent trends in food production, imports, exports, and consumption. Specifically, it examines the key factors shaping China’s food security, including demographic, environmental, and economic contexts, while proposing actionable strategies tailored to its unique circumstances. By exploring historical and current challenges alongside future projections, this study highlights the complex interactions between China’s population dynamics and food security and offers insights into sustainable pathways forward. While numerous studies have reviewed China’s food security challenges, this study specifically focuses on the pathway to achieving food security by examining key trends, policy measures, and future strategies. By synthesizing existing research and structuring solutions into an integrated framework, this review provides a comprehensive perspective on how China can sustainably enhance its food security in the long term.

## 2. Historical Population and Food Production/Consumption Projections

### 2.1. Past Predictions of Chinese Population

**Figure 1:**
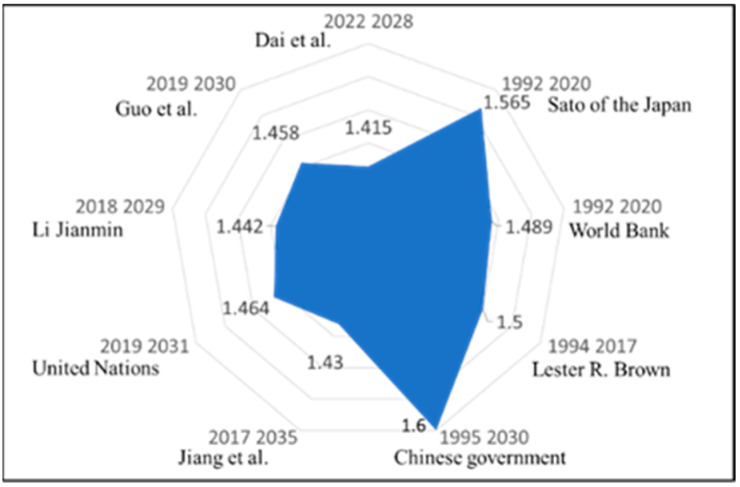
Historical population projections [24,25,26,27,28,29,30,31,32].

In 1990, China’s population was recorded at 1.134 billion. Since then, numerous projections have varied significantly among experts regarding China’s population growth trajectory. For example, as shown in Figure 1, Sato [24] of the Japan External Trade Organization (JETRO) estimated a 2020 population of 1.565 billion, while the United Nations and the World Bank projected 1.46 to 1.489 billion. Lester R. Brown [1] anticipated that the population would reach 1.5 billion by 2017, while in 1996, the Chinese government [25] projected a peak of 1.6 billion by 2030. However, more recent forecasts suggest an earlier peak and lower growth rate. The United Nations [26] estimates a peak of 1.464 billion around 2031, and Li Jianmin [27] projects a peak at 1.442 billion in 2029. Guo et al. [28] forecast a population of 1.458 billion by 2030, with a peak of about 1.46 billion by 2035. Dai et al. [29] predict a maximum population range of 1.38 to 1.45 billion, averaging a peak year around 2028. Some projections, like Jiang et al. [30] under SSP3, foresee a lower peak of 1.43 billion by 2035 under high climate stress. More recent data by Rieffel and Wang [31] indicates China’s population began to decline in 2022, and the latest United Nations report [32] suggests it could decrease to 1.3 billion by 2050. Collectively, these projections indicate a recent trend toward lower population growth rates and earlier projected peak years, with all forecasts suggesting a population peak of over 1.44 billion in China.

### 2.2. Past Predictions of Food Production/Consumption

In 1995, Lester Brown [1] voiced concerns that China might struggle to meet its rapidly rising grain demand, potentially requiring imports of 270 to 369 million tons by 2030. Brown highlighted shrinking arable land, limited irrigation resources, and the challenges in further yield improvements as key factors constraining China’s food production. In response, the Chinese government issued the “China Food White Paper” in 1996, committing to a 95% self-sufficiency rate. This optimistic stance suggested that, with strategic measures, China’s grain production could keep pace with population growth and shifting dietary habits. By 2030, China’s food demand was projected to reach approximately 640 million tons, based on a peak population of 1.6 billion and a per capita consumption rate of 400 kg. Strategies to meet this demand included boosting yields on existing farmland, developing reserve agricultural land, applying technological advancements, leveraging non-food resources, and enhancing food conservation practices.

Konishi [33] from the Japan Research Institute of Agricultural Policy anticipated substantial imports of feed grains would be needed, driven by increased demand for livestock products, industrial use, and climate challenges in key agricultural regions. He also highlighted that trade policy shifts following China’s WTO accession could impact China’s grain self-sufficiency. Overall, these forecasts reflect diverse perspectives on China’s food production trajectory, from achieving self-sufficiency to anticipating significant reliance on imports to satisfy growing consumption needs.

Many predictions of food production and consumption have been introduced (e.g., [34,35,36,37,38,39]). The peak food demand amount is predicted to be 640 to 758.17 million tons (e.g., [40]). Specially, the prediction results show that China’s grain consumption will continue to increase from 2022 to 2031, which is consistent with the factors of population change, urbanization promotion, consumption structure upgrading, and so on (e.g., [39]).

## 3. Actual Population and Food Production/Consumption Trends up to 2023

### 3.1. Changes in Population Growth

China had a population of 1.41 billion people in 2023, around 2.08 million fewer than in the previous year (not including Hong Kong, Macau, and Taiwan). Demographic growth in the country turned negative in 2022 [41,42], much earlier than previously predicted by demographers both in and outside of China. This was significantly lower than the predictions made by Lester R. Brown of the United States [1] and Mr. Sato [24] of the Asia Economic Research Institute, among others. The impact of China’s four-decade-long one-child policy and recent rapid economic development is believed to have contributed to this discrepancy. According to the “Seventh National Population Census” (National Bureau of Statistics [43]), the population distribution in 2020 was as follows: 253.38 million (17.9% of the total population) were aged 0–14, 894.38 million (63.4%) were of working age (15–59), and 264.02 million (18.7%) were aged 60 and above, with 190.64 million (13.5%) aged 65 and above. Compared to 2010, the working-age population decreased by 452.4 million (a 6.8 percentage point decrease), while the elderly population increased by 86.37 million (a 5.4 percentage point increase). The trend of a declining share of the working-age population and an increasing share of the elderly population is expected to continue, posing challenges related to population aging. Particularly in rural areas, where there has been a significant outflow of young labor, aging is more pronounced compared to urban areas. In 2020, the share of the population aged 60 and above in rural areas was 23.8%, and the share of the population aged 65 and above was 17.7%, which was 8.0 and 6.6 percentage points higher than in urban areas, respectively. Considering the delay in pension system development in rural areas, the aging issue is even more severe in rural areas than in urban areas.

### 3.2. Changes in Grain Import/Export

**Figure 2:**
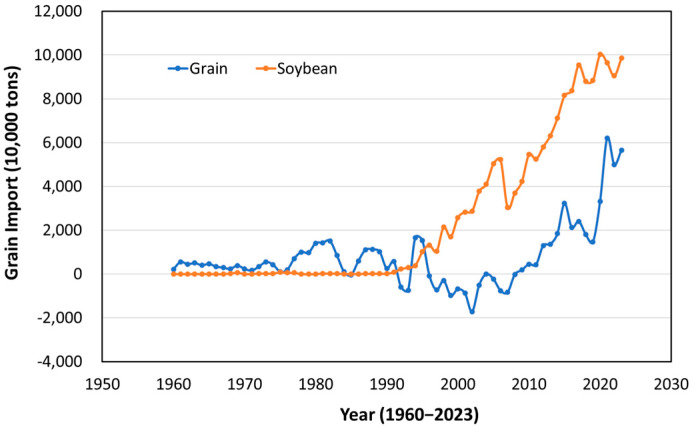
Trends in net trade volume of grains and soybeans in China (Source: National Bureau of Statistics of China, from the annual China Statistical Yearbook).

Figure 2 depicts the net trade volume of grains and soybeans in China from 1960 to 2020. Data until 2006 are from the Foreign Agricultural Policy Institute of the U.S. Department of Agriculture, and data for 2007 onwards are from Chinese statistical data [43]. It is presumed that the data from the Foreign Agricultural Policy Institute of the U.S. Department of Agriculture do not deduct exports. Grains include barley, corn, millet, mixed grains, oats, rice, rye, sorghum, and wheat. While the massive grain imports predicted by Lester R. Brown in 1995 did not materialize, China began net imports in 2009, with a growing trend observed. Particularly, soybean imports have been increasing since the 1990s, reaching 100 million tons in 2020. The total food imports (including soybeans) did not meet Lester R. Brown’s 1995 prediction but amounted to 133 million tons.

### 3.3. Changes in Food Production

**Figure 3:**
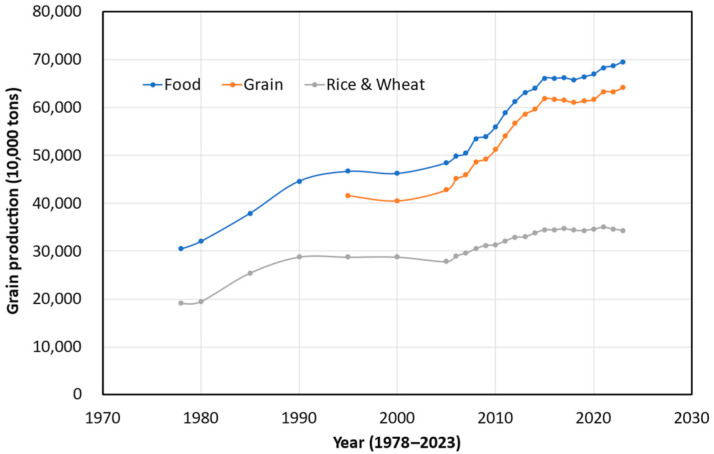
Year–on–year changes in China’s food production volume (Source: National Bureau of Statistics of China, from the annual China Statistical Yearbook).

Figure 3 illustrates the year-on-year changes in China’s food production volume. In addition to grains, legumes and tubers are included in food production. The year-on-year changes in China’s food production volume show an S-shaped curve. It gradually increased from 1978 to 1995, stagnated from the 1990s to 2005, and then experienced rapid growth from 2006 to 2016. By 2016, China’s food production volume had already reached the government’s projected production of 640 million tons by 2030. However, as shown in Figure 3, growth has stagnated in the past five years (2015–2020). Moreover, the growth rates of rice and wheat production were lower than those of other grains. The food production volume in 2020 significantly exceeded Lester R. Brown’s prediction made in 1995.

## 4. Factors Contributing to the Increase in China’s Food Production and Food Imports

### 4.1. Factors Contributing to the Increase in China’s Food Production

Based on the literature (e.g., [44,45,46,47,48,49,50,51,52,53,54]), there are the following three main factors:

#### 4.1.1. Government Food Production Policies

Kawahara [44] of the Agriculture, Forestry and Fisheries Policy Research Institute analyzed the fluctuations in China’s food production volume, identifying three corresponding periods.

(1) Price support policy (mid-1990s to 1999);
(2) Liberalization policy (2000–2003);
(3) Production subsidy policy (2004–present).

During the price support policy period, despite efforts to increase food production, surplus food resulted in stagnation due to the high prices set by the government for purchasing agricultural products. The subsequent transition to liberalization policy led to a decrease in food production as farmers’ incentive to produce decreased. However, the introduction of a production subsidy policy from 2004 onwards aimed to promote recovery and increase production through direct subsidies to farmers, continuing the foundation of market-oriented pricing and development of major production areas initiated during the liberalization policy period.

Notably, key policies such as the “National Medium- to Long-Term Food Security Strategy (2008–2020)” and the “National Food Production Capacity Increase Plan by 50 million tons (2009–2020)” played crucial roles in enhancing food production. The former emphasized domestic food supply as the cornerstone of food security, targeting a self-sufficiency rate of over 95%. The latter, based on this strategy, aimed to achieve a 50-million-ton increase in food production by 2020, focusing on 800 counties nationwide for concentrated investment to promote food production.

#### 4.1.2. Introduction of High-Yield Crops (Including Varietal Improvements) and Changes in Food Consumption Structure

Substituting low-yield crops with high-yield varieties has been crucial in increasing land productivity and food production. The introduction of hybrid rice varieties in China’s “Green Revolution” since the mid-1970s led to rapid adoption in rice-producing regions, expanding rice cultivation to northern and northeastern China by the early 1980s [45]. However, the development of new rice varieties subsequently slowed down, affecting the pace of rice production growth.

The period from 1978 to 1990 witnessed significant changes in the food consumption structure, particularly the transition from coarse grains to rice and wheat, and the introduction of high-yield crops such as hybrid rice played a significant role in this shift [46]. Varietal improvements in rice cultivation contributed to a 30% increase in rice production in China during the late 1980s to mid-1990s, with yields reaching around 9 tons per hectare by 2000. Moreover, the introduction of Super Hybrid Rice varieties since 1996 further boosted production, with yields reaching 15 tons per hectare by 2014 [47].

#### 4.1.3. Introduction of Chemical Fertilizers, Pesticides, and New Technologies

##### Chemical Fertilizers

The application of chemical fertilizers in China increased significantly from the late 1970s, particularly nitrogen, following the implementation of land privatization reforms [48]. By the 2000s, nitrogen application rates surpassed 200 kg per hectare, ranking among the world’s highest [49]. This increase in fertilizer usage significantly contributed to yield improvement, with China achieving substantial growth in grain production compared to global averages [48].

##### Pesticides

The use of pesticides also increased in China, contributing to higher food production. From 1991 to 2012, pesticide use per hectare increased by 135%, significantly surpassing the OECD average. Despite efforts to improve the safety of pesticides, China’s pesticide use per hectare remains twice the world average [50]. China has implemented a series of policies to reduce the usage of chemical pesticides to maintain food production safety and to reduce water and soil pollution. However, there is still a large gap in developing biological pesticides to replace chemical agents or managing pests to prevent crop production loss [51].

##### Introduction of New Technologies

China’s advancements in agricultural science and technology, including the development of hybrid crop varieties and modern farming machinery, have played a crucial role in increasing food production [52]. The adoption of technologies such as precision farming, drought-resistant crop varieties, and unmanned harvesting equipment has further enhanced productivity and reduced the impact of natural disasters on agricultural output [53].

In addition to technological advancements, China has actively promoted the development and commercial cultivation of genetically modified crops as a strategic measure to enhance agricultural efficiency and competitiveness [54]. The Chinese government has been actively promoting research, development, and commercial cultivation for the introduction of genetically modified crops for the past 30 years, treating it as a major national project. However, the commercialization of genetically modified crops has faced challenges due to limited varieties, low public acceptance, stringent regulations, and insufficient cooperation between research institutions and companies.

### 4.2. Factors Contributing to the Increase in Food Imports

Based on the literature (e.g., [55,56,57,58]), there are the following three main factors:

#### 4.2.1. International Grain Prices: Grain Prices Are Upside Down Domestically and Internationally

The popularity of imported grains in China stems mainly from the fact that the prices of imported grains are more favorable, particularly for some processing companies that desire to import more grains. Taking soybeans as an example, the average purchase price of domestically produced soybeans is 5500 yuan per ton, which is 2.75 yuan per 500 g. In contrast, the average price of imported soybeans in the first half of 2021 was 1.71 yuan per 500 g. Thus, the price of imported soybeans is 1.04 yuan per 500 g cheaper than that of domestically produced soybeans. From January to July 2020, the average price of imported corn was 0.87 yuan per 500 g, while the market purchase price of domestically produced corn was 1.35 yuan per 500 g, resulting in a price difference of 0.48 yuan per 500 g between domestic and imported corn. Many companies and traders opt for grain imports due to this price reversal.

#### 4.2.2. Domestic Supply Structure: The Structural Supply of Domestically Produced Grains Is Insufficient

While domestic grain production of varieties such as wheat and rice can achieve complete self-sufficiency for the nation, in some grain varieties, domestic cultivation cannot meet the market demand. Soybeans are the most obvious example, with the domestic total production in 2020 amounting to only 19.6 million tons, while market demand exceeds 120 million tons. Such significant market gaps can only be filled by imported soybeans. Additionally, grain varieties like sorghum and barley are not extensively cultivated in wide areas of China, yet there is an objective demand in the market. This is also a major reason for the significant increase in the import of sorghum and barley. Although the import volume of grain varieties like wheat remains stable, it is significantly reflected in the quality preferred by domestic consumers.

#### 4.2.3. Demand for International Trade

In addition to being market-driven, food imports can also serve diplomatic needs. For instance, when China entered the international market for high-speed railways, it obtained several resources through exchanges for high-speed railway construction projects, thereby necessitating the import of a certain amount of food. With international situations becoming increasingly complex, there might be occasions where economic trade with other countries becomes necessary, and the consumption market of agricultural products is an advantage for China. Importing an appropriate number of agricultural products can increase bargaining chips in diplomatic negotiations. For example, importing soybeans from the United States was previously China’s last resort. Therefore, some countries closely communicate with China, acknowledging that agricultural product trade is inevitable in economic cooperation. The significant increase in the import volume of grains and fruits may also be due to political factors, exceeding the expected total grain import volume.

### 4.3. Reasons Why Soybean Production Cannot Achieve 100% Self-Sufficiency

Based on the analysis of soybean production and import of China (e.g., [57,58,59]), there are three reasons, as follows.

#### 4.3.1. Low Yield of Domestically Produced Soybeans

While the yield of crops like rice and wheat commonly exceeds 600 kg/mu and sometimes even surpasses 750 kg/mu, the situation is different for soybeans. According to data released by the National Bureau of Statistics [43], the average yield of soybeans in China in 2020 was 132 kg per mu. Even in good areas, the yield per mu is only around 250 kg. The inadequate total production of soybeans in China is mainly due to the relatively low yield of domestically produced soybeans.

#### 4.3.2. Relatively High Cost of Soybean Cultivation in China

The northeastern region of China is the main soybean production area, where the planting scale is relatively large, and the level of mechanization is relatively high. However, there are several objective factors leading to increased agricultural costs such as land rents and increased agricultural expenses, including capital and labor. The planting cost is high. Compared with countries like Brazil and the United States, soybeans in China are at a disadvantage. Moreover, soybeans are clearly susceptible to natural disasters; when disasters like floods occur, the harvest of grains often fails. These factors to some extent influence the enthusiasm of farmers for soybean cultivation. In non-major soybean production areas, many farmers do not have subsidies for soybean producers, making their investment and production even more uncertain.

#### 4.3.3. Limited per Capita Land, Unable to Meet the Demand for Soybean Cultivation

While the soybean consumption demand in China continues to grow, the objective reality is that significantly increasing the domestic soybean cultivation area is difficult. Achieving complete localization of soybeans is almost impossible, primarily because there is not enough land equivalent to the cultivated area per yield of soybeans when calculated. At the current stage, even if all existing cultivated land in China were used for soybean cultivation, the total soybean production would still not meet domestic consumption. Of course, this strategy is not realistic. After all, varieties like rice and wheat are related to the staple food of ordinary people. Sacrificing staple foods to meet the market demand for soybeans is impossible. In short, the increasing popularity of imported grains reflects a certain degree of changes in market demand, and the domestic consumption level is steadily improving. However, attention should also be paid to the phenomenon of increased food imports. Ultimately, China’s approach is to avoid being controlled by others since grains are in its own hands. The challenge lies in how to effectively utilize domestic land and enhance the enthusiasm of farmers for grain production, which needs to be continuously studied.

## 5. Future Issues, Countermeasures, and Suggestions

### 5.1. Population Issue

As previously noted, China’s population growth has fallen well below earlier projections, and a clear trend has emerged of a shrinking working-age population coupled with an increasing proportion of elderly individuals, creating significant aging challenges. This issue is particularly acute in rural areas. Recognizing the seriousness of the situation, Chinese authorities have expressed a strong sense of urgency (e.g., [60,61]). As Takeshige [62] pointed out, the People’s Bank of China released a working paper titled “Understanding and Countermeasures for China’s Population Dynamics Transformation” [63]. The paper suggests various strategies, including the full removal of birth restrictions, leveraging the labor force through investments in new regions both domestically and internationally, reforming the pension system, and advancing education and technological development.

### 5.2. Food Issue

China’s population has not increased as projected, yet per capita food consumption continues to grow. Should per capita consumption remain steady, future food security concerns could be alleviated. However, with rising living standards driven by economic development, food consumption is likely to increase further.

**Figure 4:**
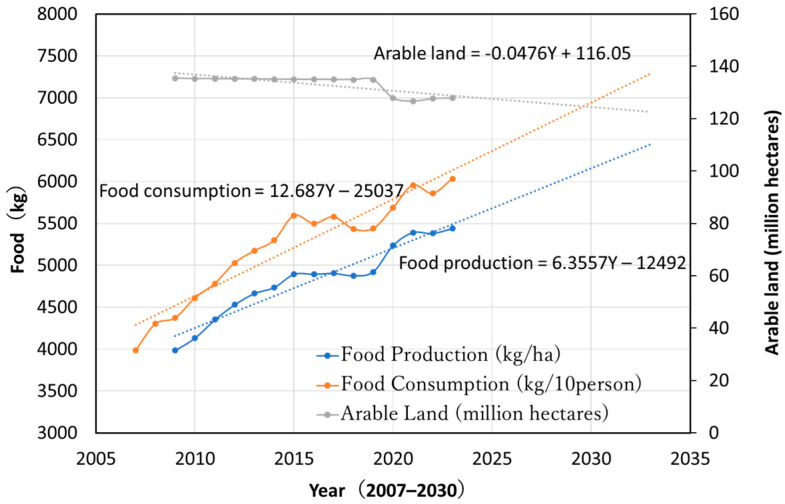
Trends and forecasts of per capita food consumption, yield per unit area, and cultivated land area in China (Source: National Bureau of Statistics of China, from the annual China Statistical Yearbook).

There are many future food issues China will face (e.g., [12,64,65,66]). The projected 15–24% reliance on agricultural imports will occur in 2030–2050. Figure 4 illustrates trends and projections for per capita food consumption, yield per unit area, and cultivated land area in China. Historical data were sourced from the “China Statistical Yearbook” published annually by the National Bureau of Statistics of China [43]. Per capita food consumption was calculated by dividing total domestic production and net imports (including soybeans) by the population, while yield per unit area was determined by dividing total domestic production by the cultivated land area. The predictions shown are based on simple regression analysis.

According to Figure 4, China’s per capita food consumption in 2023 was 603 kg per person and is projected to rise to 700 kg per person by 2030. Although this still falls short of current U.S. consumption levels, the rate of increase in yield per unit area is not keeping pace with rising consumption, and cultivated land continues to shrink. Up to 2023, yield growth per unit area has lagged behind consumption growth, contributing to an increase in China’s net food imports. As indicated in Figure 2, in 2023, China’s net food imports totaled 155 million tons, comprising 100 million tons of soybeans and 55 million tons of grains, accounting for 18.2% of total consumption. If current trends continue, food imports, including soybeans, could reach 210 million tons by 2030, representing 20% of total consumption.

Furthermore, projections for food production and consumption by 2030 (based on linear regression in Figure 4) estimate that production will reach 796.4 million tons, while consumption could rise to 1.019 billion tons (with per capita consumption at 710 kg). This would result in a deficit of 223 million tons, suggesting that imports may increase even beyond the current rate of growth.

### 5.3. Chinese Government Countermeasures

In 1996, the Chinese government published a white paper titled “China’s Food Issues” to respond to “the question of China’s future food security”. As projected, China managed its food security largely without creating major international concerns. In 2019, the government released another white paper, “China’s Food Security”, [63] which addressed various challenges, clarified its food policies, and reaffirmed its commitment and capacity to secure the nation’s food supply. This document emphasized China’s dedication to a food security strategy that reflects both domestic priorities and a global outlook. The key measures outlined include the following:

Enhancing food production capacity: protecting cultivated land and optimizing water use; ensuring stable grain-sown areas over 110 million hectares with a production capacity above 600 million tons; adjusting crop structures to increase the supply of green, high-quality grains and edible oils; stabilizing grain-sown areas while promoting region-specific crops like potatoes and beans; and advancing agricultural science to boost productivity.

Strengthening emergency grain reserve management: improving reserve management practices; enhancing the emergency food supply system; refining early warning and monitoring systems for grain conditions; and promoting conservation efforts to minimize losses.

Establishing a modern grain circulation system: accelerating the development of a modern grain market system; enhancing the construction of warehouses and logistics networks; and building a contemporary grain industry system.

Promoting global food security: actively participating in global efforts to ensure food security worldwide.

This comprehensive approach highlights China’s focus on both self-sufficiency and international cooperation in maintaining stable food supplies.

### 5.4. Some Suggestions About Food Productions

Given the trends and challenges discussed above and review all the listed studies, several strategic recommendations could strengthen China’s food production and enhance food security in the face of rising demand, demographic shifts, and environmental constraints. The proposed strategies can be grouped into four categories: policies, technology, infrastructure, and education. These categories provide a structured approach to improving food security while addressing the interconnected nature of these strategies.

#### 5.4.1. Policies

Policy interventions play a central role in guiding agricultural development and ensuring food security in China. Key policy recommendations include the following:

Promote Sustainable Agricultural Practices and Innovation: One measure is to adopt climate-smart agricultural (CSA) practices to increase resilience against climate change. CSA is crucial for enhancing agricultural resilience and mitigating climate change impacts, and CSA practices offer multiple benefits, including enhanced crop yields, farmer income, and resource efficiency [18]. Techniques like precision agriculture, water-efficient irrigation, and soil conservation should be encouraged. Encouraging these techniques is crucial for sustainable farming practices as they significantly reduce water waste, optimize resource usage, and protect the environment by minimizing soil erosion and degradation, ultimately leading to higher crop yields while minimizing environmental impact [67]. Another measure is to encourage organic and environmentally friendly farming practices to protect arable land and promote long-term soil health, particularly in regions vulnerable to degradation [18,67,68]. Another measure is to promote advanced agricultural technologies, including biotechnology, precision farming, and genetically modified crops, to boost productivity. In August 2019, Science magazine reported that China is investing heavily in crop genome editing to feed its 1.4 billion people [69]. Genetic improvements in crop varieties, such as drought-resistant or high-yield crops, could be particularly beneficial.

Protecting Farmland and Preventing Urban Encroachment: Strict enforcement of farmland protection policies is essential to ensure that sufficient arable land is preserved for agriculture. For instance, China’s “red line” policy, which safeguards 120 million hectares of farmland, has been critical in maintaining agricultural capacity (e.g., [70,71]). Such policies can mitigate the loss of productive land to urbanization and industrialization.

Enhance Support for Smallholder Farmers: Smallholder farmers account for a significant portion of China’s agricultural production. Policies providing subsidies, low-interest loans, and access to agricultural cooperatives can help enhance their productivity and resilience [72,73]. Such support ensures their integration into modern farming practices while stabilizing rural economies. A measure to ensure this is to provide financial and technical support to smallholder farmers to increase their productivity and ensure their participation in modernized agriculture.

Balancing Imports and Self-Sufficiency Goals: A measure to ensure this is to strategically import policies that can complement domestic production in order to bridge food supply gaps and import diversification and trade agreements for key crops such as soybeans and grains, which could ensure stability in supply and pricing, especially during periods of domestic shortfalls [74].

Implement Population and Labor Policies to Support Agriculture: A measure to ensure this is to address labor shortages in rural areas by creating incentives for younger generations to engage in agriculture, including policies that improve rural infrastructure, social services, and the quality of life in farming communities. Another measure is to reform retirement policies and consider programs that encourage older adults in rural areas to stay active in agriculture, possibly through flexible, part-time arrangements or community farming initiatives [75].

#### 5.4.2. Technology

Technological advancements offer transformative potential for improving agricultural efficiency and sustainability as mentioned above. In addition to these technologies, recommendations include the following:

Adopting Precision Agriculture: Technologies such as GPS-guided equipment, remote sensing, and data analytics can optimize resource use and increase crop yields. Precision agriculture not only improves productivity but also minimizes environmental impacts by reducing the overuse of inputs like fertilizers and pesticides [76,77].

Developing High-Yield and Resilient Crop Varieties: Investing in the development of genetically modified and hybrid crops that are resistant to pests, diseases, and climate stresses can significantly boost yields. For example, hybrid rice varieties have already played a crucial role in China’s agricultural success [46,78].

Utilizing Digital Agricultural Platforms: Digital platforms can connect farmers to markets, provide access to real-time data on weather and soil conditions, and facilitate financial inclusion. These tools empower farmers to make informed decisions, improving productivity and profitability [79].

#### 5.4.3. Infrastructure

Modernizing agricultural infrastructure is critical for enhancing efficiency, reducing losses, and improving overall productivity. Suggestions include the following:

Upgrading Food Storage Facilities: Developing advanced storage systems can minimize post-harvest losses and maintain food quality. Investments in grain silos, cold storage, and efficient logistics systems can ensure a stable supply chain [80,81].

Improving Irrigation and Water Management: Water scarcity is a major challenge in Chinese agriculture, particularly in northern regions. Expanding water-saving technologies such as drip irrigation and rainwater harvesting can increase efficiency and reduce water waste [82,83]. These initiatives ensure the sustainable use of water resources while supporting agricultural productivity.

Enhancing Transportation and Logistics: Building efficient food distribution networks, including rural road infrastructure and modern logistics systems, can improve market access for farmers and reduce food waste during transportation [84].

Enhance Food Import Policies as a Supplementary Strategy: Although self-sufficiency is a goal, it is necessary to ensure that food import policies remain flexible to address shortages or fluctuations in domestic production. Strategic imports of essential grains and oilseeds, like soybeans, could supplement domestic supply. A measure to ensure this is to develop trade agreements with food-exporting countries to secure a stable and diversified source of imports, ensuring food availability even in times of domestic shortfall [85].

#### 5.4.4. Education

Education and awareness are essential for building capacity among farmers and consumers, enabling them to adopt sustainable practices and reduce waste. Suggestions include the following:

Farmer Training Programs: Providing training on sustainable agricultural practices such as crop rotation, soil conservation, and organic farming can enhance productivity while minimizing environmental degradation [18,86]. Government-led agricultural extension services can play a vital role in disseminating this knowledge.

Encourage Water-Efficient Crop Choices: A measure to ensure this is to encourage the cultivation of crops that require less water, especially in water-scarce regions; promote alternative crops such as millet or sorghum where they are viable alternatives to water-intensive crops like rice and wheat; and invest in water-saving technologies and irrigation systems to optimize water usage in agriculture and reduce the dependency on groundwater and other limited water resources [87].

Promote Awareness and Education on Food Conservation: It is necessary to educate the public on the importance of reducing food waste to ensure that available food is used efficiently. Awareness campaigns such as China’s “Clean Plate Campaign” and the Anti-Food Waste Law learning on food conservation can help consumers understand their role in enhancing food security. It is important to encourage food conservation practices at all levels, from production to consumer behavior, as a way to make more effective use of existing resources for reducing food waste at all stages of consumption [88,89,90].

#### 5.4.5. Summary of the Suggestions

The proposed strategies—policies, technology, infrastructure, and education—are deeply interconnected and must be implemented in a coordinated manner to enhance food security in China. Effective policies create an enabling environment for technological advancements, while infrastructure improvements support both technological adoption and education accessibility. Meanwhile, education and training equip farmers and stakeholders with the knowledge to implement sustainable agricultural practices effectively. A holistic approach that integrates these four areas will ensure long-term food security, enhance agricultural resilience, and support China’s adaptation to climate change and evolving global food challenges.

By adopting these strategies, China can work toward a more resilient, sustainable, and efficient food production system that meets the demands of its population and economic development while minimizing environmental impact.

However, there are some limitations of this study, listed as follows:

The geographic and contextual focus on China may limit broader generalizations. The reliance on secondary data sources were synthesized to provide a comprehensive overview rather than presenting new empirical findings.

## 6. Conclusions

This review provides an in-depth analysis of China’s food security challenges, policies, and potential solutions in the context of demographic changes, environmental constraints, and evolving consumption patterns. The key findings highlight the significant role of sustainable agricultural practices, technological innovation, and targeted policy interventions in addressing food security concerns. Notably, while China has made considerable progress in boosting food production and managing imports, challenges such as shrinking arable land, an aging population, and rising per capita food consumption demand continuous attention.

The results underscore the importance of integrating practical policies and strategies to enhance food security. Strengthening the adoption of precision agriculture, water-saving technologies, and high-yield crop varieties is critical for improving productivity while reducing environmental degradation. Policies supporting smallholder farmers, protecting arable land, and advancing food storage and logistics infrastructure will further solidify China’s food systems. Additionally, strategic food imports should remain a supplementary measure, ensuring resilience against domestic shortfalls and global market fluctuations. The integration of these strategies with China’s broader rural revitalization and climate adaptation policies will ensure a holistic approach to food security.

China’s food issues remain challenging. While the Chinese government has demonstrated determination, confidence, and capability in ensuring food security, further assessment is necessary. Looking ahead, future research should focus on evaluating the long-term impact of emerging technologies, such as genetically modified crops and climate-smart farming, on food production systems. Furthermore, exploring innovative models for sustainable land use, improving rural labor retention, and reducing food waste will provide actionable insights for policy implementation. Continued collaboration between academia, government, and the private sector will be essential in translating research findings into impactful policies and practices. It should be noted that China’s food issues require radical and coordinated action by diverse stakeholders and that it is very import for the continued monitoring and adaptation of policies. By addressing these areas, China can not only secure its food supply but also serve as a model for other nations facing similar challenges in a rapidly changing world. 

## Data Availability

No new data were created or analyzed in this study. Data sharing is not applicable to this article.
